# Canine Olfaction: Physiology, Behavior, and Possibilities for Practical Applications

**DOI:** 10.3390/ani11082463

**Published:** 2021-08-21

**Authors:** Agata Kokocińska-Kusiak, Martyna Woszczyło, Mikołaj Zybala, Julia Maciocha, Katarzyna Barłowska, Michał Dzięcioł

**Affiliations:** 1 Institute of Animal Sciences, Warsaw University of Life Sciences, ul. Ciszewskiego 8, 02-786 Warszawa, Poland; Agata_Kokocinska-Kusiak@sggw.edu.pl (A.K.-K.); julia_maciocha@sggw.edu.pl (J.M.); 2 Department of Reproduction and Clinic of Farm Animals, Wroclaw University of Environmental and Life Sciences, Plac Grunwaldzki 49, 50-366 Wrocław, Poland; martyna.woszczylo@upwr.edu.pl; 3 Institute of Biological Sciences, Doctoral School, Siedlce University of Natural Sciences and Humanities, ul. Konarskiego 2, 08-110 Siedlce, Poland; mzybala@ethoplanet.com; 4 Department of Biotechnology and Nutrigenomics, Institute of Genetics and Animal Biotechnology, Polish Academy of Sciences, Jastrzębiec, 05-552 Magdalenka, Poland; k.barlowska@igbzpan.pl

**Keywords:** dogs, olfaction, behavior, chemical communication, disease detection, COVID-19

## Abstract

**Simple Summary:**

Dogs have an extraordinary olfactory capability, which far exceeds that of humans. Dogs’ sense of smell seems to be the main sense, allowing them to not only gather both current and historical information about their surrounding environment, but also to find the source of the smell, which is crucial for locating food, danger, or partners for reproduction. Dogs can be trained by humans to use their olfactory abilities in a variety of fields, with a detection limit often much lower than that of sophisticated laboratory instruments. The specific anatomical and physiological features of dog olfaction allow humans to achieve outstanding results in the detection of drugs, explosives, and different illnesses, such as cancer, diabetes, or infectious disease. This article provides an overview of the anatomical features and physiological mechanisms involved in the process of odor detection and identification, as well as behavioral aspects of canine olfaction and its use in the service of humans in many fields.

**Abstract:**

Olfaction in dogs is crucial for gathering important information about the environment, recognizing individuals, making decisions, and learning. It is far more specialized and sensitive than humans’ sense of smell. Using the strength of dogs’ sense of smell, humans work with dogs for the recognition of different odors, with a precision far exceeding the analytical capabilities of most modern instruments. Due to their extremely sensitive sense of smell, dogs could be used as modern, super-sensitive mobile area scanners, detecting specific chemical signals in real time in various environments outside the laboratory, and then tracking the odor of dynamic targets to their source, also in crowded places. Recent studies show that dogs can detect not only specific scents of drugs or explosives, but also changes in emotions as well as in human cell metabolism during various illnesses, including COVID-19 infection. Here, we provide an overview of canine olfaction, discussing aspects connected with anatomy, physiology, behavioral aspects of sniffing, and factors influencing the olfactory abilities of the domestic dog (*Canis familiaris*).

## 1. Introduction 

The sense of smell, used for chemical communication, is present in most creatures of the animal kingdom, and allows for the detection and recognition of chemical signals. Although dogs interact with their world via all of their senses, olfaction seems to be one of the most important because it provides information not only about the current status of the environment, but can also allow detection of signals from the past (presence of prey, enemies, or some new, unknown traces in the surrounding environment). This complex network of mixtures of smells creates a three-dimensional image of the surrounding world across time, playing a key role in maintaining such basic life activities as finding food, recognizing threats, or finding a reproductive partner. There is some evidence that olfaction is one of the first senses to be active, even allowing prenatal olfactory learning [1]. Canine olfaction is used in dog intra- and inter-species communication, including with man [2,3], and has been utilized by humans for over a century.

This paper presents current information about the physiological mechanisms and structures involved in canine olfaction, the behavioral aspects of sending, receiving, and interpreting smells, the factors influencing the functioning of this sense, as well as information about practical applications of canine olfaction for humans.

## 2. Anatomy and Physiology of Canine Olfaction

The canine olfactory system can recognize more smells than it has receptors for scent molecules, but olfactory receptors can have specific cross-reactions, building unique systems of patterns connected to different smells [4]. In most mammals, including dogs, there are two main parts of the olfactory system: the main olfactory epithelium (MOE) and the vomeronasal organ (VNO). The MOE is located in the usually pigmented part of the mucosa in the caudo-dorsal region of the nasal cavity, and the VNO lies between the nasal and oral cavity, near the vomer bone, just above the roof of the mouth. The nasopalatine duct, which starts behind the upper incisors on the palate, connects the mouth with the VNO, which is a tubular, elongated organ, separated by the nasal septum [5,6,7].

During sniffing, the inhaled air in the dog’s nostrils separates into two distinct pathways. The upper flow path, approximately 12–13% of each breath [8], goes straight to the olfactory region, where odor molecules are deposited and accumulate, preventing them from being exhaled. The remainder of the air, in the lower pathway, flows down the pharynx into the lungs. This path is also used for exhalation, thus supporting prolonged exposure of inspired air to the chemoreceptor area of the olfactory epithelium as air flows through the olfactory area of the dog during expiration [8,9]. Turbulence in nasal air flow is a consequence of anatomical and physiological factors. These factors influence humidification, warming, and the path of inspired air, guiding a portion of the air towards the olfactory epithelium [10]. In dogs, as in other species (e.g., human), the mechanism of nasal airflow patterns during inhalation allows the acquisition of separate odor samples in each nostril, allowing bilateral comparison of stimulus intensity and odor source localization [8].

When discussing nasal airflow, the phenomenon of sniffing lateralization observed in dogs should be mentioned. This phenomenon has been shown to be similar to auditory and visual perception in dogs [11,12,13]. Dogs have a strong right nostril bias as it is the nostril through which they first start sniffing. Then, if the smell turns out to be a familiar or non-aversive odor such as food, they shift to using the left nostril. However, if the stimuli turn out to be novel, threatening, or arousing, such as adrenaline, the dog continues to use only the right nostril. These findings are consistent with the theory reviewed by Vallortigara et al. [14,15], that the right hemisphere controls novel information processing, with the left hemisphere then taking charge of behavioral responses to familiar stimuli and the right hemisphere maintaining dominance over the sympathetic–hypothalamic–pituitary adrenal axis [16]. Behavioral lateralization directly reflects asymmetries of brain function [17], which may confer a better understanding of the training process and hence a need for new methods of training for detection dogs. As D’Aniello et al. [2] demonstrated, hemispheric specialization and chemo-signaling are also involved in the process of chemical communication between dogs and humans, allowing the transfer of information about emotional states (stress or happiness). Moreover, Webber et al. [18] proved the existence of nasal cycles in dogs in their magnetic resonance imaging study of the function of the erectile tissue surrounding the turbinates. The physiological phenomenon of the nasal turbinates’ congestion is caused by the selective activation of the autonomic nervous system by the hypothalamus. It has been hypothesized that this could assist in olfaction detection under conditions of low airflow, allowing some odors to bind to olfactory receptors [19].

The olfactory epithelium consists of olfactory receptor cells (ORCs), which are bipolar neurons extending out into the airspace to interact with odorants, as well as sustentacular and basal cells. The lifespan of an ORC is only a few weeks, and new ORCs arise from pluripotent basal cells which are capable of differentiating into either ORC or sustentacular cells [20]. Sustentacular cells (i.e., supporting cells) enwrap the ORC, lending structural support, and are involved in the phagocytosis of dead neurons and in odorant and xenobiotic substances’ transformation [20]. Among the olfactory epithelium, olfactory glands (Bowman’s glands), located in the mucosa, are responsible for the production of secretions to dissolve odorants and play a role in protection of the neuroepithelium against harmful agents reaching the nasal cavity with the collected air. In comparison to humans, dogs can detect significantly smaller concentrations of odorants due to a combination of olfactory neuron density and numbers, nasal airflow modification, and specificity of the central processing. Cilia are found on the ORC surface, with each ORC expressing only one type of olfactory receptor (OR). Proper identification of an odor seems to involve the activation of a unique combination of ORs. The number of activated ORCs is thought to be responsible for determining the intensity of the odor [21], although maximum intensity is limited and the relationship between odor intensity and the number of activated ORCs is not linear. Moreover, odor intensity depends on additional external factors such as duration of exposure to the odor and its concentration. The intensity of the odor could also be influenced by the phenomenon of adaptation [22]. In addition to neurotransmitter receptors, ORCs also have receptors for hormones, and studies performed on humans and rodents have demonstrated that odor discrimination capacities could be influenced by circulating levels of hormones (e.g., leptin or ghrelin) [23,24].

Impulses generated by olfactory cells as a consequence of odor detection impulses are transmitted, through the cribriform plate of the ethmoid bone, by the olfactory nerves (creating the cranial nerve 1-CNI). The structure that constitutes the next level of the olfactory pathway is the olfactory bulb (OB), which is located under the frontal lobes [25]. The OB contains glomeruli created from bundles of nerve fibers and is the place where incoming receptor cell axons contact by the synaptic connection with the dendrites of mitral neurons, and axons transfer impulses to the other areas in the brain. The OB plays both a modulatory and a sensory role. It is involved in the initial processing and filtering of olfactory information, allowing discrimination between odors, as well as enhancing the sensitivity of odor detection and filtering out background odors [21,26]. Compared to other senses, where the sensory track is crossed, olfactory pathways lead ipsilaterally from the detection area located in the nasal cavity to the perception area in the brain, which means that the right nostril is the source of signals for the right brain hemisphere, and receptors located in the left nostril transmit impulses to the left hemisphere.

From the OB, olfactory signals are transmitted to the olfactory cortex, containing the anterior olfactory cortex, piriform cortex, peri-amygdaloid cortex, and entorhinal cortex. The first three areas transmit the olfactory signal to the frontal cortex and thalamus, while the entorhinal cortex sends the impulses to the hippocampal formation, which is involved in memory recognition of odors [26]. The structure responsible for the odor thresholding is supposed to be the thalamus [26].

**Figure 1:**
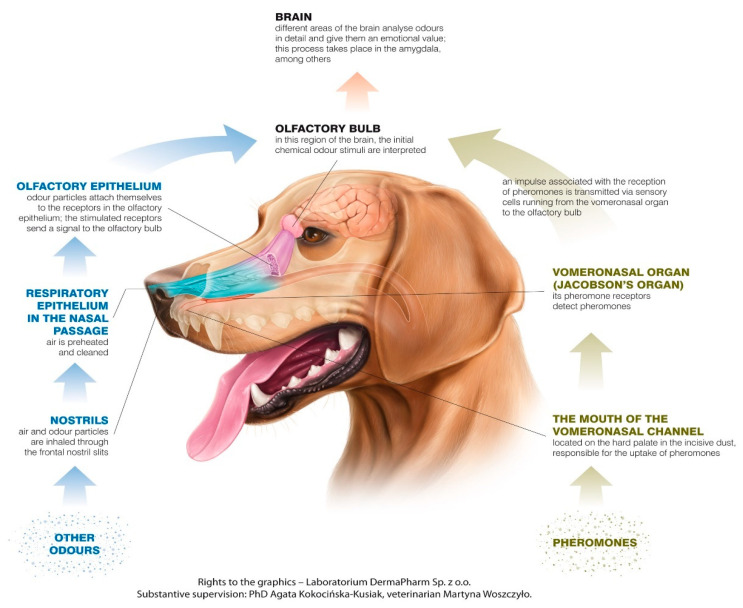
This schematic shows the traditional structure of the chemical substances detection system. Currently, the distinction between the so-called nose and the VNO in terms of the detection of odors and pheromones is not so obvious, and researchers tend to believe that both the “nose” receives pheromone signals and the VNO receives stimuli, with low-volatility odorant compounds suspended in the liquid phase.

The MOE and the VNO are physiologically independent in the collection of smell signals and the specific signals to which they react [7]. Signals perceived by the MOE and VNO are also separated in the pathways leading to the brain [27]. They can be used in different tasks to perceive different kinds of stimuli—the VNO is not only the main structure in pheromone recognition, it can also be used in recognizing other low-volatile substances [28] (Figure 1).

In comparison to some other species, the canine VNO lacks a VR2-based vomeronasal subsystem, as confirmed by the observation that the canine system does not express Gαo and expresses Gαi2 throughout the AOB as well as in the VNsE, and by the lack of functional VR2 genes in the canine genome [6,29].

While many other species of mammals have additional organs involved in chemoreception, such as Masera’s Septal Olfactory Organ, which has been recognized in cat fetuses [30], these have not been detected in dogs [30,31]. Similarly, the Grüneberg ganglion, which supports both chemo- and thermo-sensing in other species, is often, erroneously, shown to be a part of the canine chemoreception system, but has not yet been identified in dogs. Although this ganglion might possibly be present in the early embryonic stages, it seems to regress during prenatal development [32]. 

In the human, as Jacquot et al. [33] stated, nasal detection of volatile chemicals is mediated both by the olfactory and trigeminal systems. Additionally, Kobal et al. [34] suggested that that in the human, odors can only be detected when the olfactory stimulants simultaneously excite the trigeminal somatosensory system. However, Jenkins et al. [21] indicate that even though some odors can stimulate the nasal mucosa trigeminal nerve endings (detecting feelings such as warmth or coolness), in dogs, odor detection is implemented only through the olfactory neuroepithelium. 

## 3. Internal and Environmental Factors Influencing Olfactory Skills: Olfaction Gene Polymorphism, Age, Sex, and Breed-Specific Olfactory Capacity

### 3.1. Genetic Implications

The largest mammalian gene superfamily is connected with the olfactory receptors. While in the human more than 50% are recognized as pseudogenes, only about 20% of OR genes are functionally inactive in dogs. The percentage of pseudogenes, as well as the frequency of specific gene polymorphisms, varies by breed, which could be one of the reasons for breed-specific olfactory capacity. It seems, therefore, that innate genetic factors may contribute to the occurrence of genetically determined lines of dogs with greater suitability for olfactory work [21]. 

### 3.2. Breed

The olfactory receptor (OR) genes form the largest gene families in mammalian genomes. Taking into account this large gene pool and the huge variety of dog breeds, it was worth investigating the connection between olfactory capability and breeds. The genetic evaluation performed by Tacher et al. [35] within and between dog breeds focused on the gene polymorphism of the first element of the olfactory pathway—the olfactory receptors. They found that “some alleles are breed-specific (or rare in the dog population), with some representing the major allele in the breeds concerned”. 

In their study, Robin et al. [36] correlated a high overall level of polymorphism with the receptor’s ligand-binding capacity, suggesting that this phenomenon could be responsible for differences in the potential for particular breeds to act as sniffer dogs.

However, the results of the research concerning the determination of the most olfactory-sensitive breeds are not consistent, with different authors indicating in their observations that different breeds are the most predisposed to the olfactory work [21,37,38,39,40,41]. These results could suggest that in addition to genetic predisposition, some behavioral attributes, such as inherent motivation, eagerness to learn, trainability, and ability to work with people, could also significantly influence general canine olfactory capability. 

An appreciation of these two factors is thought to be at the root of the creation of Sulimov dogs, a jackal–dog hybrid breed with unique olfactory abilities. The dog breeds used in the original development of this hybrid (Lapponian Herders, Nenet Herding Laika, Fox Terrier, and Spitz) were selected with the aim of improving trainability [7]. This seems to confirm once again that in the utilization of canine olfactory skills, the behavioral aspect is equally as important as the purely genetically determined olfactory ability. 

### 3.3. Age and Sex

Similar to the other senses, olfactory capabilities can decrease with age due to atrophic changes, with degeneration observed in the olfactory epithelium and fewer olfactory cells in aged dogs [42]. Moreover, in comparison with juvenile dogs, older individuals may have a much stronger long-term memory of odors and can deal with more complicated odor information [43].

In the context of differences of olfactory capabilities between genders, the results of Wei et al. [43] indicate that cells in the olfactory bulbs of female dogs are more active than those in males. 

### 3.4. Environmental Conditions

With regard to the effect of environmental conditions, humidity has been found to be an important factor in improving olfactory skills in dogs, probably due to improved nasal humidity and odorant trapping [21]. Moreover, according to Gutzwiller [44], increased humidity could be responsible for increased odor intensity, positively influencing the tracking efficiency of dogs. This phenomenon is also thought to be true in the context of semiochemical substance detection [45]. However, even though the increased humidity connected with light rain is usually perceived as a positive influence on odor detection, heavy rain is usually a negative factor, since it could force the scent down lower to the ground [46]. Humidity associated with foggy weather can also be a negative factor, causing scents to hang in the air and forcing the dog to scan the entire area, slowing down the process of tracking. 

Higher temperatures can also negatively influence canine olfaction. Bräuer and Blasi [46] showed that this was due to a reduced ability of dogs to work, causing a decline in searching performance [46]. However, beyond the direct influence of temperature on working ability, there is also the risk of dehydration as a consequence of increased activity in hot conditions, which can reduce canine olfactory efficiency due to decreased enzyme activity and nasal mucosal fluidity [21].

Moreover, it is important to take into account that environmental conditions can change throughout the day, and that seasonal changes, especially transitional periods (such as melting snow revealing freshly sprouting vegetation), can also influence the efficiency of the sniffing dogs. Changes in the physical environment, such as sudden landform changes (e.g., where open ground changes into woods, or farmland where fertilizers or pesticides have been freshly applied), can also offer a further complication [47]. 

Among environmental factors that could influence the sniffing performance (olfactory acuity), Jenkins et al. enumerates factors such as diet components negatively/decreasing (coconut oil) or positively/increasing olfactory acuity (corn oil, EPA, DHA, DPA, animal-based proteins) [21]. Generally, it can also be concluded that olfactory work, which is a combination of physical and mental work, requires large amounts of energy which must be provided in the diet.

### 3.5. Diseases 

Improper air flow, affecting olfactory skills, could be caused by diseases such as nasal cavity tumors, local injuries, or specific infections such as canine distemper or parainfluenza. In the case of hyposmia in dogs caused by endocrinological disorders such as hyperadrenocorticism, hypothyroidism, or diabetes, a neural mechanism is probably involved, similar to the anosmia observed in some cases of COVID-19 infection in humans [48,49].

In the context of semiochemical substance detection, inflammatory and degenerative disorders have been suggested as possible causes of VNO dysfunction in cats and pigs [50,51]. In dogs, Dziecioł et al. [5], in a paper dedicated to the issue of evaluation of the MRI features of the canine VNO, mentioned an MRI head examination method as the basis for the development of VNO in vivo diagnostics and the possibility of detection of pathology in animals with behavioral problems influenced by improper semiochemical communication between inter-individuals.

### 3.6. Substances and Drugs Influencing Olfactory Abilities

The influence of drugs on the sense of smell could be connected with a loss of acuity [52] and/or distortion of function (dysosmia). The mechanisms responsible for those events are thought to be connected with inhibition of odorant receptors. As Henkin [52] suggested, this effect can be achieved by inducing abnormal persistence of receptor activity or by blocking receptor activation. Taking into account the number of drugs used in canine veterinary medicine, available studies have tested a less than representative number of substances for effects on canine olfaction. Even though zinc sulfate [53] has been used routinely in various studies for destruction of the main olfactory epithelium in many species [53], Ramaihgari et al. [54] found that that zinc nanoparticles could potentially be used to increase canine detection capabilities in environments with very low concentrations of the odorants. Among the drugs used for anesthesia and analgesia in dogs, isoflurane and propofol, as well as fentanyl followed by naloxone, have been investigated in terms of olfaction capability in dogs, but no negative effect on canine olfaction was observed [55,56]. Similarly, a study performed in humans revealed no influence of oral hormonal contraceptives on olfaction [57]. However, some studies have identified medications that had a negative effect on canine olfaction. Jenkins et al. [56] found that oral administration of metronidazole (25 mg/kg every 12 h) degraded the ability of working dogs to detect the odors of explosives. Similarly, the use of steroids (dexamethasone or hydrocortisone) caused a significant elevation in the olfactory detection threshold of dogs, without any observable structural alteration of the olfactory tissue using light microscopy [58]. 

## 4. Olfactory Behavior

### 4.1. Sniffing vs. Smelling

Understanding the physiological process of olfactory detection requires discrimination between two processes: sniffing and smelling. In this context, smelling is an implicit (unconscious), effortless, non-cognitive process that is an accompaniment of breathing, facultatively bringing the stimulus to the dog’s attention. In turn, sniffing could be defined as an explicit [26], effortful, cognitive behavior.

The “sniff cycle” might play a role in odor coding, by allowing the timing of spikes with respect to the phase of the respiration cycle to encode information about odor identity or concentration [59]. Sniffing can also be dynamically coordinated with rhythmic neural activity, which indicates the coordination of the olfactory system with other brain areas [59]. However, sniffing is usually used as an ongoing, continuous rhythmic process, allowing the environment to be scanned, with even a single sniff being enough for accurate discrimination between very similar odors [59]. Sniffing is also thought to be combined with the ability to localize odors [60]

At the level of the brain, “sniffing whether odorant is present or absent, induces activation primarily in the piriform cortex of the temporal lobe and in the medial and posterior orbito-frontal gyri of the frontal lobe”, while a smell, “induces activation mainly in the lateral and anterior orbito-frontal gyri of the frontal lobe” [61]. 

In the study of Berns et al. [62], the activation of the caudate nucleus of the canine brain during fMRI examination was evaluated using un-sedated and unrestrained dogs. They reported activation of the olfactory bulb when the dogs were presented with a scent collected from familiar or unfamiliar humans or dogs. However, maximal activation of the caudate region only occurred when the scent of a familiar person was presented. 

### 4.2. Sending and Receiving Olfactory Signals 

One of the most important behaviors in dogs’ olfactory communication is sniffing conspecific individuals. In neutral situations, males spend more time than females in sniffing the rear parts of the partner, especially the area close to the anal glands [63], while females seem to concentrate more on the head area during olfactory exploration [64]. Sniffing is mutual and both individuals are collecting information [65]. 

However, even without direct contact, dogs can communicate with each other through chemical signals [66]. Urine-marking, which involves leaving urine or other body secretions on distinctive elements of the landscape [67], is the most common form of scent marking [68]. The marked object, such as a tree or a streetlamp, becomes a scent message containing much information about the individual who left it [65]. Adult males typically use the raised-leg posture, leaving an elevated mark, juvenile males use the lean-forward posture during which no hind limb is raised, and most females use the squat posture [69]. Male dogs and wolves mark more frequently in unfamiliar areas and will continue to do so even if their bladder is empty, implying that marking is not associated with urine passage [70]. Males urinate more frequently and direct more urinations onto vertically oriented targets than females [71], and small dogs urinate more frequently than large dogs, probably because scent marking involves less risk than direct interaction [68].

Overmarking [72], where one individual places its scent mark on top of the scent mark of another individual, may happen in various situations. Competitive countermarking is used by both sexes, although males overmark more than females, and is strongly connected with social status, suggesting that dogs might be able to assess the status of unfamiliar dogs by olfactory cues in their countermarks [73]. Overmarking occurs with the same frequency in intact and castrated dogs [74], and high-status dogs investigate more urine marks than low-status dogs [73]. Overmarking female urine by males might happen to guard potential mates, and this might be enforced by aggressive encounters and domination display [75]. Overall, countermarking might just be a way of exchanging information [65], allowing individuals to assess competitors and to evaluate potential mates [73], but smaller dogs can exaggerate their signals and their competitive ability by using a higher leg raise during urination. Dunbar [64] reported that male dogs sniff the urine of females longer, which means that some odors are more interesting than others.

Although wolves use feces for territorial marking [70], defecation is thought to play a less important role in communication in dogs [69]. However, when walked by an unfamiliar man, shelter dogs defecated less than when walked by an unfamiliar female [76].

Scent-rubbing, when an individual sniffs the source of an exceptionally fragrant odor and then drags the head or shoulders through it, followed by the back and rump, is a widespread behavior in carnivores. Several explanatory theories have been attributed to this behavior, extensively described by Horowitz [65] and Allen et al. [77]. Some individuals use it as a camouflage, disguising themselves by covering their own smell to hide by blending into the environment or blending into a group. This last behavior is often used by individuals changing groups—they rub themselves in the alpha male’s feces. Others use it as a means of transferring important information to their family group, or to elevate their own social position by carrying the most desirable odor, or simply as a hedonistic pleasure; however, no consensus has been established.

### 4.3. Tracking Behavior

A set of psychophysical features is responsible for the suitability of dogs to detect objects and people [78]. This competence is strongly connected to the dog’s ability to detect, discriminate, and identify stimuli [66]. Thesen et al. [79] described in detail the unique dogs’ tracking behavior, well-known to the dog owners. In their work, they distinguished three phases: (1) an initial searching phase, during which the dog tried to find the track, (2) a deciding phase, during which it tried to determine the direction of the track, and (3) a tracking phase, in which it followed the track. The second phase is significantly longer than the others, but only five sequential footsteps are enough for a dog to determine the direction [80]. 

Taslitz [81] distinguished two main methods of tracking. The first was ground sniffing, when the dog registers odor molecules left on the ground and follows the scent trail with its nose close to the ground, ignoring distracting stimuli, until the source of the scent is found. The second was air sniffing with the nose up, where the olfactory molecules move with the movement of the air, to detect airborne scents in large-area searches where there is no scent trail to follow [82]. Dogs can use either or both of these methods, depending on the weather conditions [83], the environment in which they are working [62], the scent they are looking for [82], and training [41]. 

In favorable conditions, against the wind, dogs can detect the smell of a wild tortoise from up to 60 m away [84], a rodent in the natural environment up to 50 m [85], decaying meat up to 200 m [86], and whale scat up to 1.93 km [87]. They are even able to detect live leverets—a target difficult to detect olfactorily [88].

Some of the main limiting factors of detection and tracking performance are weather conditions, such as temperature, wind, and humidity [83,89], and breed type, as breeds that had been originally selected for scent work demonstrate a higher olfactory acuity than those not selected for such work [41]. Although breeds with a high number of odor receptor cells and elongated noses with a large nasal cavity, for example Bloodhounds [90], might be expected to perform better than brachycephalic dogs, which have a smaller olfactory bulb [91] and disturbed nasal air passage [92] that lowers their ability to track a scent [41], Hall et al. [93] suggest that differences connected with the breed specificity do not exist at all. Other factors include the age and type of scent, as some scents are easier to recognize and some decay faster [94], as well as methods of training [39,95,96]. 

Some of the ways that sniffing differs from breathing include the frequency of air sampling, olfactory flow rate, and odorant uptake [97]. Behavioral observation, such as duration of sniffing, were used successfully to assess olfactory performance in detection dogs, especially in differentiating true from false-negative responses, which might impact the most crucial parts of the work carried out by detection dogs, when a human life is at stake [98].

## 5. The Use of Canine Olfactory Skills

The sensitivity of canine olfaction is closely connected with the structure of their olfactory system, which is both quantitatively and qualitatively very different to that of humans (for example, dogs have more ORCs and a larger OB, allowing for enhanced sensitivity of odor detection) [99,100]. This is why it is difficult to predict exactly how dogs perceive their environment and how to adjust the techniques of human–dog cooperation to obtain the best results. Until humans have a full understanding of the potential of canine olfaction, the possibility remains that in using dogs’ sense of smell for work, the limits still lie in humans’ perception and learning, rather than in the dogs’ olfactory system.

### 5.1. Detection of Dangerous and Illegal Substances

Among dogs working as sniffer dogs globally, an important group are those working in the detection of explosives [7,101]. The use of trained animals, not only dogs but also other species such as Giant African Pouched Rats, is considered to be a very reliable, versatile, and cost-effective method of explosives’ detection as they are better than specialized devices at discriminating and locating target scents while ignoring all other interfering scents present in the environment [102]. It is estimated that there are 100 million landmines scattered all around the world, blocking access to agricultural lands and restricting economic growth, as well as killing and injuring people [103]. Service dogs locate buried landmines and are used to declare an area mine-free [104,105]. Thanks to their training, they can find the explosive materials and chemicals used in producing the mines [106,107]. The effectiveness of detector animals in the identification and management of explosive materials is hard to overestimate [102,108]. 

In terms of their quality of work in detecting explosives, a very important factor is that during their training, dogs are taught to locate the residual scent of flammable products used to catalyze combustion, as well as ignoring scents of burnt carpets, wood, or other products of pyrolysis [109]. Dogs are also much better and more precise than humans in detecting traces of accelerants among burnt debris, as they can register and trace amounts of scents down to 5.0 to 0.005 μL, which compares to the level of sensitivity of laboratory techniques [110,111,112].

Dogs can also be trained to identify areas contaminated with dangerous substances such as toluene [113]. They can detect these substances in very small amounts (0.1 g) and over long distances, where conventional equipment fails. This increases human safety by identifying the outermost borders of affected areas without exposure to dangerously high levels of toxins and enables the determination of source points for more efficient sampling of the contaminants [113]. Detection of organochlorines in exported Australian beef was made possible through dogs that are now routinely employed in the detection of aldrins, dieldrin, and DDT contamination in arable land. The levels of contaminants in the soil can be as low as 1 ppm or less, and trained dogs still have 99% accuracy in detecting the point sources of organochlorine compounds. Therefore, using dogs saves time and reduces the number of samples needed to identify contaminated areas [114].

Customs officers and border controls utilize trained service dogs in detecting illegal drugs such as cocaine, heroin, methamphetamine, and marihuana, and are routinely employed in screening millions of people and goods crossing international borders in airports, marine ports, and mail depots [115,116,117]. 

### 5.2. Detection of Biological Scents

Dogs are being used more often in the detection of biological scents, such as human odor, as they can isolate and recognize the odor of a specific person from amongst other persons or even when the odor is mixed with other, stronger scents [118]. The police in some countries utilize dogs’ olfactory abilities to identify criminals, by matching scents found at the crime scene with the odor of the accused. Although this approach is controversial, and its credibility is questioned in many studies, some police forces still regard this as one of the most valuable jobs a police dog can perform. Scientific results show that after appropriate training, dogs can match odors from different parts of the same human body [119,120,121]. Moreover, dogs can trace a path of human odor through very busy city centers, up to 48 h after they have been created, with an average accuracy of 77.5% [122].

In search and rescue, dogs are being used to find victims of all sorts of events: avalanches, earthquakes, floods, plane crashes, etc. [123,124]. A separate group of service dogs, trained to detect decomposing human bodies, are often used at accident scenes [125]. Some subsets of these dogs, called cadaver-detection dogs, can find traces or remains of human bodies such as bones, bodily fluids, and tissue, both above- and below-ground as well as in the water [123,125]. These dogs operate with a detection efficiency in the range of 30% to 81% in field trials and can cover a large area quickly, thus saving emergency services and law enforcement both time and effort [125,126].

### 5.3. Detection of Other Living Organisms

Preventing the accidental spread of the Brown tree snake (*Boiga irregularis*) in Guam is one example of dogs being used in biological safety and border control, with an average detection effectiveness of 62% [127,128,129]. Dogs’ detection abilities are also used in agriculture, such as for locating plant parasites such as the palm weevil [130], which massively affects the most important crop in the Middle East, Date palm (*Phoenix dactylifera* L.) plantations [130]. As reported by Nakash et al. [130], it is very important to discover palm weevil invasion very early, and the two dogs that were trained to detect secretions from affected trees achieved a very high detection rate in the initial testing. Wallner and Ellis [131] reported an average detection success rate of 73% for two dogs trained in reacting to masses of eggs of gypsy moths [131] located close to the ground, in the ground, or in the forest litter, and very difficult to find. The results also show a strong correlation between one dog’s number of detections and the density of the egg masses found [131]. In the USA, dogs are achieving 95% accuracy in detecting eastern subterranean termites [132], which can cause close to two billion dollars in damage and pest control, annually [133]. The damage can occur quite early in the infestation, before its discovery, as visual detection in this initial phase is very often impossible [132]. The dogs trained to find the termites at the early stages of development can discriminate between them and other burrowing insects (i.e., ants, cockroaches) as well as identify wood damaged by the termites [132]. Compared to electronic odor detectors, dogs detecting western subterranean termites (Reticulitermes hesperus Banks) achieved a 98% success rate in identifying artificially placed colonies, while human-made devices achieved a much lower percentage [134]. However, dogs returned 28% false-positive detections when invasion did not take place, which can be attributed to imperfect training techniques [132]. Specially trained dogs were also shown to have a very high success rate (99.7%) in detecting screw-worm fly in both infected slugs and wounds on large animals infected via slugs [135]. This fly can kill warm-blooded animals and cause serious economic losses [135]. 

Interestingly, trained dogs can even detect microorganisms. Some cyanobacteria in commercial catfish farms produce compounds that accumulate in the fish flesh, causing an unpleasant smell [136]. The cost of discarding affected fish stock is between 15 and 23 million US dollars in the USA alone [137], as cited in [136]. Shelby et al. [136] showed that dogs can identify 2-methylisoborneol and geosmin in the pond water with high accuracy. Three dogs detected the smells with an accuracy of 79% to 92% at the level of 1 μg/L and an accuracy of 37% to 49% at concentrations of 10 ng/L. According to Shelby et al. [136], trained dogs can offer an alternative method to chemical analysis and a more practical way of detecting unpleasant smells. The growth of mold and other fungi in the building can have detrimental effects on peoples’ health and can cause costly deterioration of the building. Detecting these microorganisms early is extremely difficult. Kauhanen et al. [138] tested the effectiveness of dogs trained to detect mold and other fungi within construction materials, and found that two dogs were able to identify 75% of all hidden samples containing microorganisms. 

Dogs can also be helpful to conservation scientists when trained to find endangered wild animal species. Due to the low density and vast areas these species can potentially occupy, it is often very difficult to keep track of them. So-called ‘Scat dogs’ are trained to find and follow the scats of a given endangered species. As Wasser et al. [139] report, this non-invasive method of searching for animal scats can increase the number of samples collected by decreasing the false identification of scats. The information gathered from scats is comparable to data derived from traditional methods. Molecular analysis of scats delivers data on the species, sex, diet, parasites, and the individual character of the animal [140,141]. Breeding and stress hormones can indicate the fertility and reproductive rate as well as the influence of any disorders on the physiological state [139,142]. By collecting the scat samples systematically over a large enough area, an estimate of the population characteristics can be drawn, such as sex balance, kinship, niches, and range [139,140]. Scats can also offer a better source of DNA samples than hair, skin, feathers, nails, bones, or saliva [140]. According to Wasser et al. [139], an animal distribution derived from scat searching performed by dogs overlaps with the distribution derived using other methods, such as collecting hairs or tracking using GPS transmitters.

In North America, dogs are being used to manage and protect the population of game animals [143], and grizzly bears (*Ursus arctos horribilis*) in Canada, described by Wasser et al. [139]. The dogs were trained to find bear scats over 500 km^2^, with DNA studies used to determine the species and identify individual bears. Similar methods have been used to assess the bear population in the whole of North America [143].

Scat dogs are also used in studies of a very rare subspecies of kit fox called the San Joaquin (*Vulpes macrotis mutica*) in the USA [144]. The difficulty for dogs and humans in these studies is that the scats of the target fox have to be identified from other, very similar scats left by coyotes (*Canis lurrans*), skunks (Mephitis mephitis), and badgers (Taxidea taxus). The study showed that dogs were capable of 100% accuracy in identifying the species, up to four times the success rate when compared to trained human trackers [144,145]. This ability of scat dogs is so much more valuable, as using DNA extraction from scats and determining species using laboratory methods is very expensive. In Russia, the same principle is used to identify other species as well. Dogs are trained to match urine samples collected in the wild to the sample in the data library. A combination of traditional tracking and dogs’ detection is used to monitor the movements of individual tigers [146], but data on the population dynamics of tigers is collected using dogs only. Two dogs used in this study showed 89% and 96% accuracy in detecting tigers that were new to the study area [146]. Dogs used in traditional bird hunting are now being used to locate and study those very endangered bird species [147]. Researchers are using the characteristics of certain dog breeds and their congenital hunting skills in rare species management programs. For example, a Border collie was used to catch the Aleutian cackling goose (*Branta Canadensis leucopareia*) in order to relocate them to predator-free islands in Alaska [148]. When compared to humans doing the same capture task, dogs were far less likely to injure themselves or the ducks, as well as being more efficient. Scientists managed to catch 120 geese in 3 weeks, while only 2 trained dogs caught 143 birds within just 4 days [148]. In New Zealand, dogs have been used for over 100 years to locate many endangered species such as Kiwi bird (*Apteryz* spp.), Kakapo (*Strigop habroptilus*), or Blue duck (*Hymenolaimu malacorhynchos*) [149,150]. Possibilities for using the canine sense of smell seem to be unlimited.

## 6. Recognition of the Physiological State by Olfaction

The recognition of the features of the physiological state of other individuals is an important skill in the context of social interaction. These skills could be divided into unlearned, occurring spontaneously, and learned (often during specialized training). The first group would include the use of olfaction to recognize species, gender, and age, as well as physiological condition, such as phase of the reproductive cycle and emotions [2,151].

### 6.1. Detection of the Phase of Reproductive Cycle

It has been observed that some stud dogs can distinguish between particular phases of heat in bitches, and will attempt mating only with females that are at the so-called optimal time for mating—a behavior that some breeders used to use for identification of this period instead of using a laboratory test based on progesterone level determination [151,152]. Moreover, dogs were also able to detect changes in the odor of a female in estrus treated with antibiotics [153].

Dogs can identify dairy cows in heat by the scent of their vaginal fluid, urine, milk, and blood plasma at an accuracy of 78% to 99% [154,155]. Dogs can also distinguish milk from a cow that is in proestrus, estrus, and diestrus phase [156]. 

### 6.2. Recognizing Emotional State

As a synanthropic species, the dog is good at dealing not only with the visual expressions of emotion but also with interspecies chemo-signaling. The study of D’Aniello [157] proved that dogs easily recognize human emotions, such as fear or happiness, by olfaction. Moreover, it has been observed that human odorant stress signals provoked a longer reaction in dogs than happiness signals, and generally, this reaction was not gender-dependent [157]. Semin et al. [158] suggested two possible variants of the mechanism responsible for this phenomenon. The first is that volatility has a distinct chemical composition that activates emotion-specific behaviors and physiological states. The second pointed to an associative learning process, indicating that the dogs are able to identify a distinct odor that is emitted with a distinctive behavioral syndrome.

There are also reports of dogs being used to predict aggressive outbursts in psychiatric patients using vision and olfaction. However, although their accuracy in detecting upcoming aggression attacks was 100%, the dogs were not always able to point out a particular patient [159]. What this type of study shows is a proof-of-concept that dogs can work with a large group of unfamiliar people and be able to detect, among those people, unifying and universal scents or other criteria-meeting stimuli.

### 6.3. Dogs Detecting Diseases in Humans and Animals

The volatilome, understood as the composition of both the volatile organic compounds (VOCs) in an organism and the VOCs reflecting its unique current metabolic state (including the influence of infection), can be used to detect diseases and the presence of specific pathogens. Those substances released in concentrations of ppb to ppt [160] and ppm to ppb (human blood and urine) are certainly within a dog’s capabilities of detection, since dogs’ sense of smell demonstrated a lower limit of detection at concentrations of one part per trillion (ppt) [161].

Upon the observation of spontaneously presented behaviors when dogs recognize some disturbances in the physiological state of another individual (e.g., human), people start to reinforce these behaviors and train these abilities. In the case of seizure in humans, there was a lot of doubt that dogs could recognize specific changes in the odor of the person, or warn the owner based on subtle changes in behavior that are imperceptible to humans [162]. However, current studies have shown that seizures are associated with olfactory-specific characteristics and can be easily detected with high accuracy by trained dogs [163]. Additionally, in the case of narcoleptic patients, it has been confirmed that trained dogs can detect patients’ distinct typical odor [164]. However, Wells et al. [165] and Weber et al. [166] report that behavioral reactions to hypoglycemic episodes in pet owners with type 1 diabetes commonly occur even in untrained dogs [167], suggesting that dogs can be trained to detect hypoglycemic breath of an individual human. 

The presence of the malaria parasite changes the smell of an infected person, making him more attractive to mosquitoes, and the study dedicated to the issue of recognition of the changes in the smell of these persons confirmed the ability of trained dogs to detect the specific skin odor marker for this disease [168].

Dogs were also trained to recognize diseases in exhaled air, urine, feces, and cancer tissue samples from patients affected with different types of tumors: lung [160,169,170,171,172,173], breast [169,174], prostate [175,176], ovary [177], bladder [178], and large intestine [179], and distinguish these samples from those taken from healthy patients. Dogs spontaneously detected melanoma developing in their owners and were able to identify not only melanoma developing on the patient’s skin, but also detect cancer cells which were placed on the skin surface of healthy patients [180]. 

Detection of viral infections, such as COVID-19, by smell is not connected with the specific scent of the infectious agent, in this case the virus, but based on substances released by infected cells. The characteristic scent is strongly connected with the air exhaled by individuals infected with the virus. In exhaled air, VOCs are in the gas phase, and non-volatile molecules are included in liquid phases, such as exhaled breath condensate (EBC) and aerosols (EBA) [181]. Humans infected by SARS-CoV-2 were studied with multi-capillary column-coupled ion mobility spectrometry [182], which can detect SARS-CoV-2 infection and Influenza A infection in the breath [182]. In scientific reports, dogs were trained to detect samples collected from patients diagnosed with COVID-19 and distinguish them from samples collected from healthy patients. The average diagnostic sensitivity in one of the studies was 82.63%, with 96.35% specificity, and the overall average detection rate achieved by the dogs was 94% (±3.4%). The dogs were presented with 1012 randomized samples, and correctly indicated 157 samples as positive and 792 samples as negative, with 33 samples incorrectly indicated as negative and 30 samples incorrectly indicated as positive [183]. More scientific examples of dogs sniffing COVID-19 were presented by Grandjean et al. [184], whose team successfully performed a proof-of-concept study including dogs detecting sweat samples from patients infected with this virus. In one of the most recent publications in this field, which used exhaled breath samples, a detection accuracy of >90% and positive predictive values ranging from ~73% to 93% were reported for four dogs within their first month of training [185]. Although, as for cancer-detecting dogs, the applicability of disease detection methods based on the canine sense of smell can be controversial, the authors hope that this kind of approach can still offer one more layer of protection in the fight against SARS-CoV-2. What seems to be especially interesting is that specific VOCs can be distinguished from viral infection caused by different viruses, which could be important in the context of similarity of clinical symptoms observed, for example, during influenza and coronavirus infection [186]. In that study, the authors determined that VOCs produced during infection with three live influenza virus subtypes were unique for each virus subtype [186]. In the context of the differentiation of pathogens based on the VOCs they produce, Abd El Qader et al. [187] proved that the VOCs produced during bacterial and viral infection can also be distinguished. Interestingly, Mashir et al. [188] proved that not only natural infection, but also vaccination using an attenuated intranasal vaccine against influenza A (H1A1), caused the huge number of changes in exhaled breath, which could suggest that changes in odors are connected strictly with the activation of the immune system. Currently, studies on the possibility of using dogs to distinguish between various viral infections of the human respiratory tract are ongoing.

Even though the canine sense of smell has been used mostly as a tool for human disease detection, it has also been used successfully in the field of animal disease detection, although with a much more restricted range of application. Alasaad et al. [189] describe the use of dogs trained to detect the scent of Sarcoptes-infected animals in the Alps. Amongst other effects, Sarcoptes infection causes the formation of thick crusts on the skin, which can easily become infected and emit a strong specific odor, which can be detected and recognized by humans in close proximity. However, even in the difficult alpine environmental conditions, it was still possible for the trained dogs to detect infected animals, allowing the identification, separation, and capture of animals with mange.

Trained dogs have also been used to differentiate between nematode-infected and uninfected sheep feces. In that study, three species of nematodes were used in the training of the dogs, resulting in a mean success rate of 76–80% for the detection of a particular parasite species, and a 92% reliability rate in the detection of mixed infections [190].

Another study reported a male German wirehaired pointer being trained to detect screwworm (Cochliomyia hominivorax)-infested animals, with a success rate of 100% (265 tests) with training dummies and 94.7% (18 successes for 19 tests) with screwworm-infested animals [135].

Although dogs’ capabilities to detect various diseases have been demonstrated in numerous studies, it still seems to be a controversial or even doubtful method. Thus, in some studies, researchers have tried to combine these methods with a “traditional” analytical approach, such as VOC analysis, which can identify specific disease biomarkers. This model is thought to be useful because although the disease-detecting dogs (DDD) do not detect these particular chemical compounds, we believe that they will still detect markers specific for the disease substances. Combination of DDD with analytical methods allows the identification of relevant biomarkers or just confirmation of the differences in the chemical profile between healthy and sick individuals [185].

## 7. Chemical Communication Influencing Animal Behavior

In all these aspects of human use of canine olfactory ability, the final results, understood as an indication or identification of the odor (chemical signal), depend on the ability of the handler to recognize the specific behavioral signals presented by the animal. Those signals could be spontaneous or learned. This aspect of signaling the detection of a chemical marker seems to be especially important in the context of detection of substances that are naturally stimulating to dogs, such as canine sex pheromones, because in this case, the signs of natural arousal are the most desired reaction. That also seems to be important in the context of realizing what exactly the dog detects and points to—the substance of interest itself, or an accompanying substance. In the study of Jezierski et al. [191], dogs were able to detect and point to samples collected from a female in estrus with satisfactory and significant accuracy. However, they did not usually present any signs of expected behavior—in this case, arousal. This seems to be surprising, since the reaction towards pheromones is supposed to be characteristic, repetitive, and more importantly, subconscious, and therefore difficult to inhibit. In that study, the full range of behavior exhibited during contact with urine samples collected from a female in estrus was only observed when samples were placed in the imitation recto-vaginal region of an artificial dog model. This observation suggests that the behavioral reaction of dogs towards chemical signals can be modified by other environmental elements, which could provide the context. In other species, for example, ungulates and horses, easily visible specific behavior—the flehmen response—makes it easier to evaluate the behavioral reaction to the semiochemical signaling. Thus, the probable coexisting involvement of two systems: MOB and AOB (connected to the collection of the samples of chemical markers into VNO), makes this model of detection more complex and more difficult for evaluation of dogs’ behavior.

Although the dog is a predator species, it can also become the prey in some circumstances. In that case, detection of the odor of the predator (bear or lynx) could result in modification of the dog’s behavior. What is interesting is that this behavioral reaction towards the secretions of potentially dangerous individuals can be identified spontaneously, in the absence of a previous bad experience. Samuel et al. [192] mentioned that although chemical cues (such as urine, feces, fur, and anal gland secretions) usually cause modification of animal behavior as an experience-based reaction, interspecific reactions enabling prey to detect adverse cues from predators that they have never previously been in contact with have been documented. It is worth mentioning that in this case, the observed behavioral parameter is not increased interest (expressed by longer sniffing time) but rather avoidance behavior of the area marked with a particular odor.

Many different physiological and behavioral parameters used to be included when evaluating the influence of the various chemical signals on the signal-receiver’s behavior. In dogs, the most common parameter, after adopting a specific learned body position or vocalization (e.g., detection of explosive materials, drugs, and samples containing cancer tissue), is sniffing time [191,192,193]. Heart rate or blood flow in particular organs can also be used as markers of chemical signal detection, especially in the case of subconscious responses, such as those with pheromones or predator scent detection [152,192]. Since behavioral reactions are very often ambiguous and difficult to interpret, new methods to evaluate the impact of odors on the chemical signal-receiver are still being proposed. In cases where a strong behavioral reaction is not advisable, such as during EEG or fMRI studies requiring long immobilization, dogs must be trained to not react to the odors presented, since reaction of the particular brain regions is interpreted as a reaction to the olfactory signal [62,193,194].

## 8. Novel Methods of Canine Olfaction Evaluation—fMRI Study

When the sense of smell as well as semiochemical communication began to be studied in humans using modern methods of brain function visualization, the idea of a similar study in dogs arose. As in humans, a detailed fMRI evaluation of the reaction of the dog brain to odors confirmed the activation of those regions of the brain previously suggested to be responsible for odor detection. However, it should be mentioned that most of these studies specifically focused on those particular areas of the brain, thus activation of other regions of the brain could potentially have been missed. Another potentially useful approach is the resting state examination protocol (RS fMRI), which has been used to map regional interactions in the brain in the absence of a task. Although the first of this type of study in both un-sedated and sedated dogs has been published, it did not include an olfaction evaluation, as was performed in human studies [195,196].

One of the advantages of fMRI is the possibility of obtaining a detailed evaluation of the brain’s reaction to a mixture of odors or to identify the particular brain region(s) activated by odors connected with some features (e.g., familiar or unfamiliar odor), studies that would be difficult to carry out using traditional, behavioral methods of examination [194].

In Berns et al.’s [62] study, their observation of caudate activation allowed them to draw the conclusion that dogs could not only discriminate a familiar scent among others, but also had a positive association with it.

Another advantage of using functional brain visualization methods is that they could offer the possibility of evaluating the influence of a low odor concentration, that might be undetectable consciously, but which could still alter brain activation, confirming its subconscious detection [197]. The use of brain visualization necessarily excludes the simultaneous evaluation of behavioral events, which could by misinterpretation (e.g., overinterpretation or unnoticed) lead to false conclusions. However, this kind of method still has its own limitations that could also lead to the improper interpretation of results. The necessity of using specially trained dogs is another disadvantage of this method. Thus, in the future, a combination of the more traditional, behavioral methods, together with the sophisticated methods of brain activity visualization, will be required.

## 9. Limitations in Canine Detection—A Critical Assessment

Although a number of publications and studies have confirmed the high applicability of the canine sense of smell in many fields, it must also be pointed out that all methods using dogs as detectors still have limitations, and reports of low efficiency, false positives, as well as false negatives, have unfortunately been taken as an indication of limited usefulness, overshadowing some quite spectacular achievements The key problem in using dogs for detection is that they are living animals, so there are a number of variables that can influence their performance, including their state of health (illness, pain) which can affect both their physical and mental condition (agitation, fearfulness, aggression), as well as mood, lifestyle, diurnal rhythm, stressful situations, traumatic experiences at work, psychophysical activity, rest, sleep, circadian rhythm, sexual cycle, and many more.

All these types of factors affect every living organism, and thus can potentially create artefacts. In addition, there is a training factor. The learning process is continuous, so going through training does not necessarily mean that a dog will remember all the skills it acquired during training (therefore, it is important to remind the dog of specific commands), and it is also constantly learning new skills. In addition, the selection of training methods is crucial for the dog’s future skills. Sometimes, training produces a useful effect, and other times quite the opposite. Therefore, dogs that were trained to detect narcotics were beginning to show, for example, various types of foils and adhesive tapes, which are very often wrapped around drugs. Thus, they generalized labeling responses to other, accompanying odors. In addition, in connection with their desire to obtain reinforcement, dogs often give false positives when trying to force this attention. Proper training and regular controlled application of the scent significantly reduce this type of behavior, but do not completely eliminate it.

An issue which should always be considered during training, as well as during task performance, is the avoidance of sample contamination. Contamination of the sample (possibly during improper collection, storage, and handling) is a very serious mistake, responsible for the false results when it is not well-recognized. In such a case, the error attributed to the dog was instead a methodological error, for which the person who incorrectly carried out the sample preparation procedure was responsible. Even samples that were technically collected following the same procedure can still vary in their scent background (e.g., samples collected at two different hospitals in which different disinfectants are used or samples collected from the patients residing at home). Although it has been suggested that the amount of the substance or the sample size could also influence the level of detection, Lazarowski et al. stated that beliefs that the amount of odor available can be easily altered by increasing the mass are not correct, because the amount of odorant emitted from a given substance is also related to the substance’s vapor pressure and other factors, including environmental temperature, humidity, etc. [96]. Jezierski et al. identified that individual elements such as style and time of sniffing can influence the results of a dog’s olfactory work [78]. Other factors such as the effects of the dog’s routine, the use of cues other than olfactory ones (visual), and the non-verbal communication between handler and dog have also been mentioned [39,71,72,73,74,75,76,77,78,79].

Even though Gazit and Terkel [101] showed that explosive-detecting dogs mostly used their sense of smell to detect their target and that the lack, or intensity, of light did not influence the results, the question remains whether dogs trained to detect targets by olfaction always rely only on their sense of smell or whether they use additional strategies such as guessing [198] or visual information. Some studies have indicated that, contrary to general expectations, dogs do also rely on visual factors [198,199,200].

However, although visual stimuli might often help a dog to properly identify the target, under some circumstances, it can be a disturbing factor and can even worsen the animal’s concentration [201].

In the field of disease detection, despite the numerous studies presenting a high efficiency of the sniffing dogs, a serious problem with the generalization of a cancer odor during robust double-blind tests involving new samples has been reported [176]. In the case of diabetes-alerting dogs (DADs), the conclusion that adoption of this kind of animal can be beneficial to the owner suffering from diabetes has been discussed in a few studies that highlighted numerous doubts regarding their effectiveness and the mechanism of detection (i.e., what the dogs are actually detecting) [202,203,204]. Los et al. [202] emphasized the high false-positive rate observed in their study, while Gonder-Frederick et al. also complained about a low accurate detection level in both low and high blood glucose events [203].

Hall and Wynne [205] reported in turn that dogs trained using odor mixtures tend to perform better in detection tasks than when trained on pure odors. These findings highlight the potential limitations of training dogs to detect a specific target odor while expecting them to indicate to the target when it is mixed with distractors. That also shows the influence of the method of training on the results of odor detection, with the authors concluding that dogs could not spontaneously recognize a target component in an odor mixture after target-only training. Among the elements influencing the final results of olfactory work of the sniffing dogs is the issue of an animal’s ability to respond to odors based on prior odor training [206]. In this context, Dorman et al. [206] showed that dogs trained with different forms of ammonium nitrate were unable to recognize the substance with an acceptable level of success when it was mixed with powdered aluminum, with the results from some animals no different to those expected by chance alone.

Lazrowski and Dorman [207] showed that 87% of dogs trained to detect potassium chlorate (PC) as a component of explosive material did not correctly signal the presence of one or more PC-based explosive mixtures out of a sample size of four.

Another important element in the consideration of dogs’ olfactory skills and the effectiveness of odor detection is that many studies only used a small group of animals (sometimes as few as even one or two), and so generalization of the results to the sniffing dogs’ population can be confusing. In their review of methodological approaches, Johnen et al. [208] reported that in 14 studies involving sniffing dogs, the average number of dogs per study was 4.6, with a range of 1 (*n* = 5) to 10 (*n* = 2). 

A complex, critical review of the various factors influencing canine odor detection skills has been presented by Hayes et al. [209]. The combination and interrelationships of canine physiology and training methodologies, as well as factors such as cultural, legal, and scientifically established domains, were identified as elements which should be taken into account when discussing the usefulness of dogs as detectors. There was also some attention paid to the issue of the relationship between dog and handler and factors such as the canines’ ability to recognize their handler’s facial expressions. The role of the handler in a successful olfactory detection process could also be connected with the rate of positive reinforcers (rewards) offered to the dog. The motivation of a sniffing dog could also decrease when objectives were less likely to be found. The issue of the dog’s motivation is also thought to be an important element because dogs work for the reward, and over time they could try to find easier ways to obtain a reward, for example by trying to guess or pointing to the target by chance, etc. Thus, continuous training and verification of the accuracy of detection is a necessary, indispensable element in maintaining the detection capabilities of dogs detecting odors.

A very interesting concept related to the bilateral interaction between handler and sniffing dogs has been proposed by Lit et al., who postulated that the handler’s beliefs affect the outcome of scent detection by the dog [210]. A strong relationship between the dog and handler could also be the reason for the lower effectiveness of odor detection when a handler preferred by the dog is replaced by another; apart from the increased risk of misunderstanding within the team, the dog could also experience decreased motivation [209].

Limitations related to the use of dogs as odor detectors can also be seen when comparing the results of scientific studies with their practical application in the field, particularly in dogs used as disease detectors. A good example is in dogs trained to detect cancer. Even though their detection efficiency has been confirmed in dozens of publications, this method has still not been adopted as a standard procedure in human healthcare. It is possible that a similar fate awaits this method of detecting COVID-19 infections, although work is currently underway on this issue in many countries. One of the most important issues that can influence the final results of a study involving sniffing dogs are the methodological tools used for the preparation of the experiment as well as for the evaluation of results. While variations in the features of dogs, handlers, and the environment are sometimes, or even often, difficult to avoid, a different methodological approach can sometimes surprise and significantly influence the final results and conclusions. Johnen et al. in their study pointed out that creating a methodologically correct protocol is a major challenge that not all authors of studies are able to effectively overcome [211]. They also regret that “no accepted quality standards or adequate guidelines for performing scent studies and testing dogs have been published in peer-reviewed literature yet”. The heterogeneity of the methods used affects those authors’ opinions of the possibility of using inappropriate tools and thus obtaining false conclusions, but evaluation of the relevant literature has demonstrated an increase in the number of experiments using the same methodology [208,211]. To avoid methodological errors, some researchers propose the use of devices that allow automated data collection to reduce or even eliminate the subjectivity and unintended reinforcement delivery [212].

In the creation of a proper environment for a study, it is very unlikely that all potentially disturbing factors can be avoided, especially those related to sample volatility, therefore if the task is performed in the field, all of these factors must be recorded rather than controlled.

## 10. Conclusions

Dogs have been supporting people around the world for years by searching for various types of odors. The potential of using the canine’s sense of smell is very wide and is still developing in new directions.

**Figure 2:**
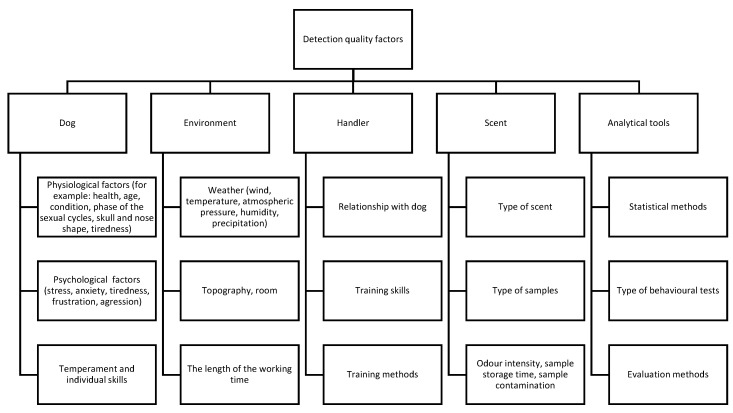
Factors affecting the efficiency of canine olfactory detection.

Overall, the mechanism of olfactory detection and the ensuing innate canine olfactory abilities make them more sensitive detectors than the best man-made analytical instruments. However, some issues make it an instrument whose credibility is constantly subjected to doubt, and so its use is not as widespread as might be expected. The most important issues influencing the efficiency of the results of canine olfactory work are presented below (Figure 2).

The biggest problem with the objective assessment of canine detectors is that as a living organism, they are constantly changing, depending on the external environment. It must be understood that the use of canine olfactory skills can only be helpful if the biological character of the “detector” is always considered, and the handler is familiar with both the dog’s personality and other aspects of canine physiology. The final results of this teamwork can be affected if the dog is experiencing boredom, fatigue, lack of appropriate stimuli, no bond with the handler, or handler insensitivity to the signals sent by the dog, in addition to other factors such as health status or age [21].

On the other hand, the issue of proper design of scientific experiments as well as a practical training schedule and daily dog work may reduce the risk of mistakes made by dogs and humans. For this purpose, reliable, detailed described procedures must be developed. They must include elements such as using as many dogs as possible (preferably at least 5), the use of sample randomization, the use of new samples in testing (other than training samples) that dogs have never smelled before, double-blind testing, appropriately selected and clearly presented statistical methods, and objective discussion of the results [213].

However, there are some issues that cannot be eliminated. These include, among others, the individual characteristics of both the dog and the handler, which are not subject to standardization procedures (the outstanding talent of a dog or handler cannot be reduced, and the smaller skills of other teams working at 100% of their abilities cannot be increased). This represents a large difference from analytical devices which always follow standardization procedures.

The compilation of the laboratory methods related to the olfactory skills of odor-detecting dogs is one approach that would improve the efficiency of detection of searched substances, and could even offer a step towards the identification of the particular compounds or the collection of sufficient data for additional discrimination between positive and negative samples [185,211].

## Data Availability

Not applicable.

## References

[1] Hepper P.G., Wells D.L. (2005). Perinatal Olfactory Learning in the Domestic Dog. Chem. Senses.

[2] D’Aniello B., Semin G.R., Alterisio A., Aria M., Scandurra A. (2018). Interspecies transmission of emotional information via chemosignals: From humans to dogs (*Canis lupus familiaris*). Anim. Cogn..

[3] Miklosi A., Topál J., Csányi V. (2007). Big thoughts in small brains? Dogs as a model for understanding human social cognition. NeuroReport.

[4] Stitzel S.E., Aernecke M.J., Walt D.R. (2011). Artificial Noses. Annu. Rev. Biomed. Eng..

[5] Dzięcioł M., Podgórski P., Stańczyk E., Szumny A., Woszczyło M., Pieczewska B., Niżański W., Nicpoń J., Wrzosek M.A. (2020). MRI Features of the Vomeronasal Organ in Dogs (*Canis familiaris*). Front. Veter. Sci..

[6] Salazar I., Cifuentes J.M., Sanchez-Quinteiro P. (2012). Morphological and Immunohistochemical Features of the Vomeronasal System in Dogs. Anat. Rec. Adv. Integr. Anat. Evol. Biol..

[7] Jezierski T., Ensminger J., Papet L.E. (2016). Canine Olfaction Science and Law: Advances in Forensic Science, Medicine, Conservation, and Environmental Remediation.

[8] Craven B.A., Paterson E.G., Settles G.S. (2009). The fluid dynamics of canine olfaction: Unique nasal airflow patterns as an explanation of macrosmia. J. R. Soc. Interface.

[9] Settles G.S., Kester D.A., Dodson-Dreibelbis L.J., Barth F.G., Humphrey J.A.C., Secomb T.W. (2003). The External Aerodynamics of Canine Olfaction, in Sensors and Sensing in Biology and Engineering.

[10] Patel R.M., Pinto J.M. (2014). Olfaction: Anatomy, physiology, and disease. Clin. Anat..

[11] Siniscalchi M., Quaranta A., Rogers L.J. (2008). Hemispheric Specialization in Dogs for Processing Different Acoustic Stimuli. PLoS ONE.

[12] Siniscalchi M., Sasso R., Pepe A.M., Vallortigara G., Quaranta A. (2010). Dogs turn left to emotional stimuli. Behav. Brain Res..

[13] Siniscalchi M., Sasso R., Pepe A.M., Dimatteo S., Vallortigara G., Quaranta A. (2011). Sniffing with the right nostril: Lateralization of response to odour stimuli by dogs. Anim. Behav..

[14] Vallortigara G., Rogers L., Bisazza A. (1999). Possible evolutionary origins of cognitive brain lateralization. Brain Res. Rev..

[15] Vallortigara G., Chiandetti C., Sovrano V.A. (2011). Brain asymmetry (animal). Wiley Interdiscip. Rev Cogn. Sci..

[16] Craig A.D. (2005). Forebrain emotional asymmetry: A neuroanatomical basis?. Trends Cogn. Sci..

[17] Siniscalchi M., D’Ingeo S., Quaranta A. (2016). The dog nose “KNOWS” fear: Asymmetric nostril use during sniffing at canine and human emotional stimuli. Behav. Brain Res..

[18] Webber R.L., Jeffcoat M.K., Harman J.T., Ruttimann U.E. (1987). MR Demonstration of the Nasal Cycle in the Beagle Dog. J. Comput. Assist. Tomogr..

[19] Sobel N., Khan R.M., Saltman A., Sullivan E.V., Gabrieli J.D. (1999). Olfaction The world smells different to each nostril. Nature.

[20] Liang F., Fengyi L. (2020). Sustentacular Cell Enwrapment of Olfactory Receptor Neuronal Dendrites: An Update. Genes.

[21] Jenkins E.K., DeChant M.T., Perry E.B. (2018). When the Nose Doesn’t Know: Canine Olfactory Function Associated with Health, Management, and Potential Links to Microbiota. Front. Veter. Sci..

[22] Sirotin Y.B., Shusterman R., Rinberg D. (2015). Neural Coding of Perceived Odor Intensity. eNeuro.

[23] Karlsson A.C., Lindroos A.K., Lissner L., Torgerson J.S., Carlsson B., Carlsson L.M.S., Sjöström L. (2002). Evidence for gender-specific associations between leptin and olfaction. J. Gender-Specific Med. Off. J. Partnersh. Women’s Health Columbia.

[24] Loch D., Breer H., Strotmann J. (2015). Endocrine Modulation of Olfactory Responsiveness: Effects of the Orexigenic Hormone Ghrelin. Chem. Senses.

[25] Kavoi B.M., Jameela H. (2011). Comparative Morphometry of the Olfactory Bulb, Tract and Stria in the Human, Dog and Goat. Int. J. Morphol..

[26] Jia H., Pustovyy O.M., Waggoner P., Beyers R.J., Schumacher J., Wildey C., Barrett J., Morrison E., Salibi N., Denney T. (2014). Functional MRI of the Olfactory System in Conscious Dogs. PLoS ONE.

[27] Swaney W., Keverne E.B. (2009). The evolution of pheromonal communication. Behav. Brain Res..

[28] McGann J.P. (2017). Poor human olfaction is a 19th-century myth. Science.

[29] Young J.M., Trask B.J. (2007). V2R gene families degenerated in primates, dog and cow, but expanded in opossum. Trends Genet..

[30] Kociánová I., Gorošová A., Tichý F., Čížek P., Machálka M. (2006). Structure of Masera’s Septal Olfactory Organ in Cat (*Felis silvestris* f. catus)—Light Microscopy in Selected Stages of Ontogeny. Acta Vet. Brno.

[31] Ma M., Fleischer J., Breer H., Eisthen H.L. (2015). The Septal Organ, Grueneberg Ganglion, and Terminal Nerve. Handbook of Olfaction and Gustation.

[32] Barrios A.W., Quinteiro P.S., Salazar I. (2014). Dog and mouse: Toward a balanced view of the mammalian olfactory system. Front. Neuroanat..

[33] Jacquot L., Monnin J., Brand G. (2004). Influence of nasal trigeminal stimuli on olfactory sensitivity. Comptes Rendus Biol..

[34] Kobal G., Van Toller S., Hummel T. (1989). Is there directional smelling?. Cell. Mol. Life Sci..

[35] Tacher S., Quignon P., Rimbault M., Dreano S., André C., Galibert F. (2005). Olfactory Receptor Sequence Polymorphism within and Between Breeds of Dogs. J. Hered..

[36] Robin S., Tacher S., Rimbault M., Vaysse A., Dréano S., André C., Hitte C., Galibert F. (2009). Genetic diversity of canine olfactory receptors. BMC Genom..

[37] Hall N.J., Smith D.W., Wynne C.D. (2015). Pavlovian conditioning enhances resistance to disruption of dogs performing an odor discrimination. J. Exp. Anal. Behav..

[38] Jezierski T., Adamkiewicz E., Walczak M., Sobczyńska M., Górecka-Bruzda A., Ensminger J., Papet E. (2014). Efficacy of drug detection by fully-trained police dogs varies by breed, training level, type of drug and search environment. Forensic Sci. Int..

[39] Lazarowski L., Rogers B., Waggoner L.P., Katz J.S. (2019). When the nose knows: Ontogenetic changes in detection dogs’ (*Canis familiaris*) responsiveness to social and olfactory cues. Anim. Behav..

[40] Lazarowski L., Waggoner L.P., Krichbaum S., Singletary M., Haney P.S., Rogers B., Angle C. (2020). Selecting Dogs for Explosives Detection: Behavioral Characteristics. Front. Veter. Sci..

[41] Polgár Z., Kinnunen M., Újváry D., Miklosi A., Gácsi M. (2016). A Test of Canine Olfactory Capacity: Comparing Various Dog Breeds and Wolves in a Natural Detection Task. PLoS ONE.

[42] Hirai T., Kojima S., Shimada A., Umemura T., Sakai M., Itakurat C. (1996). Age-related changes in the olfactory system of dogs. Neuropathol. Appl. Neurobiol..

[43] Wei Q., Zhang H., Ma S., Guo D. (2017). Sex- and age-related differences in c-fos expression in dog olfactory bulbs. Acta Zool..

[44] Gutzwiller K.J. (1990). Minimizing Dog-Induced Biases in Game Bird Research. Wildl. Soc. Bull. (1973–2006).

[45] Majumder S.S., Bhadra A. (2015). When Love Is in the Air: Understanding Why Dogs Tend to Mate when It Rains. PLoS ONE.

[46] Bräuer J., Blasi D. (2021). Dogs display owner-specific expectations based on olfaction. Sci. Rep..

[47] Shrestha S., Kamel F., Umbach D.M., Freeman L.E.B., Koutros S., Alavanja M., Blair A., Sandler D.P., Chen H. (2019). High Pesticide Exposure Events and Olfactory Impairment among U.S. Farmers. Environ. Health Perspect..

[48] Myers L.J., Nusbaum K.E., Swango L.J., Hanrahan L.N., Sartin E. (1988). Dysfunction of sense of smell caused by canine parainfluenza virus infection in dogs. Am. J. Veter. Res..

[49] Myers L.J. (1990). Dysosmia of the dog in clinical veterinary medicine. Prog. Vet. Neurol..

[50] Asproni P., Cozzi A., Mainau E., Temple D., Manteca X., Bienboire-Frosini C., Pageat P. First description of vomeronasal organ inflammatory changes in pigs. Proceedings of the Annual Congress of the European College of Animal Welfare and Behavioural Medicine (ECAWBM).

[51] Asproni P., Cozzi A., Verin R., Lafont-Lecuelle C., Bienboire-Frosini C., Poli A., Pageat P. (2016). Pathology and behaviour in feline medicine: Investigating the link between vomeronasalitis and aggression. J. Feline Med. Surg..

[52] Henkin R.I. (1994). Drug-induced taste and smell disorders. Incidence, mechanisms and management related primarily to treatment of sensory receptor dysfunction. Drug Saf..

[53] Keller M., Douhard Q., Baum M.J., Bakker J. (2006). Destruction of the Main Olfactory Epithelium Reduces Female Sexual Behavior and Olfactory Investigation in Female Mice. Chem. Senses.

[54] Ramaihgari B., Pustovyy O.M., Waggoner P., Beyers R.J., Wildey C., Morrison E., Salibi N., Katz J.S., Denney T.S., Vodyanoy V.J. (2018). Zinc Nanoparticles Enhance Brain Connectivity in the Canine Olfactory Network: Evidence from an fMRI Study in Unrestrained Awake Dogs. Front. Veter. Sci..

[55] Essler J.L., Smith P.G., Berger D., Gregorio E., Pennington M.R., McGuire A., Furton K.G., Otto C.M. (2019). A Randomized Cross-Over Trial Comparing the Effect of Intramuscular Versus Intranasal Naloxone Reversal of Intravenous Fentanyl on Odor Detection in Working Dogs. Animals.

[56] Jenkins E.K., Lee-Fowler T.M., Angle T.C., Behrend E.N., Moore G.E. (2016). Effects of oral administration of metronidazole and doxycycline on olfactory capabilities of explosives detection dogs. Am. J. Vet. Res..

[57] Schaefer M., Iravani B., Arshamian A., Lundström J.N. (2021). No Evidence That Hormonal Contraceptives Affect Chemosensory Perception. i-Perception.

[58] Ezeh P.I., Myers L.J., Hanrahan L.A., Kemppainen R.J., Cummins K.A. (1992). Effects of steroids on the olfactory function of the dog. Physiol. Behav..

[59] Kepecs A., Uchida N., Mainen Z.F. (2005). The Sniff as a Unit of Olfactory Processing. Chem. Senses.

[60] Frasnelli J., Charbonneau G., Collignon O., Lepore F. (2008). Odor Localization and Sniffing. Chem. Senses.

[61] Sobel N., Prabhakaran V.T., Desmond J.E., Glover G.H., Goode R.L., Sullivan E.V., Gabrieli J.D.E. (1998). Sniffing and smelling: Separate subsystems in the human olfactory cortex. Nature.

[62] Berns G.S., Brooks A.M., Spivak M. (2015). Scent of the familiar: An fMRI study of canine brain responses to familiar and unfamiliar human and dog odors. Behav. Process..

[63] Preti G., Muetterties E.L., Furman J.M., Kennelly J.J., Johns B.E. (1976). Volatile constituents of dog (*Canis familiaris*) and coyote (*Canis latrans*) anal sacs. J. Chem. Ecol..

[64] Dunbar I.F. (1977). Olfactory preferences in dogs: The response of male and female beagles to conspecific odors. Behav. Biol..

[65] Horowitz A. (2016). Being a Dog: Following the Dog into a World of Smell.

[66] Gadbois S., Reeve C., Horowitz A. (2014). Canine Olfaction: Scent, Sign, and Situation. Domestic Dog Cognistion and Behaviour: The Scientific Study of Canis familiaris.

[67] Hart B. (1974). Environmental and hormonal influences on urine marking behavior in the adult male dog. Behav. Biol..

[68] McGuire B., Bemis K.E. (2017). Scent marking in shelter dogs: Effects of body size. Appl. Anim. Behav. Sci..

[69] McGuire B., Olsen B., Bemis K.E., Orantes D. (2018). Urine marking in male domestic dogs: Honest or dishonest?. J. Zool..

[70] Harrington F.H. (2006). Double Marking in Arctic Wolves, Canis lupus arctos: Influence of Order on Posture. Can. Field-Nat..

[71] McGuire B. (2016). Scent marking in shelter dogs: Effects of sex and age. Appl. Anim. Behav. Sci..

[72] Ferkin M.H., Pierce A. (2007). Perspectives on over-marking: Is it good to be on top?. J. Ethol..

[73] Lisberg A.E., Snowdon C.T. (2011). Effects of sex, social status and gonadectomy on countermarking by domestic dogs, *Canis familiaris*. Anim. Behav..

[74] Kaufmann C.A., Forndran S., Stauber C., Woerner K., Gansloßer U. (2017). The Social Behaviour of Neutered Male Dogs Compared to Intact Dogs (*Canis lupus familiaris*): Video Analyses, Questionnaires and Case Studies. Veter. Med. Open J..

[75] Cafazzo S., Natoli E., Valsecchi P. (2012). Scent-Marking Behaviour in a Pack of Free-Ranging Domestic Dogs. Ethology.

[76] McGuire B., Fry K., Orantes D., Underkofler L., Parry S. (2020). Sex of Walker Influences Scent-marking Behavior of Shelter Dogs. Animals.

[77] Allen M.L., Gunther M.S., Wilmers C.C. (2016). The scent of your enemy is my friend? The acquisition of large carnivore scent by a smaller carnivore. J. Ethol..

[78] Jezierski T., Walczak M., Górecka A. (2008). Information-seeking behaviour of sniffer dogs during match-to-sample training in the scent lineup. Pol. Psychol. Bull..

[79] Thesen A., Steen J.B., Døving K.B. (1993). Behaviour of dogs during olfactory tracking. J. Exp. Biol..

[80] Hepper P.G., Wells D.L. (2005). How Many Footsteps Do Dogs Need to Determine the Direction of an Odour Trail?. Chem. Senses.

[81] Taslitz A.E. (1990). Does the Cold Nose Know—The Unscientific Myth of the Dog Scent Lineup. Hastings Law J..

[82] Beebe S.C., Howell T.J., Bennett P.C. (2016). Using Scent Detection Dogs in Conservation Settings: A Review of Scientific Literature Regarding Their Selection. Front. Veter. Sci..

[83] Jinn J., Connor E.G., Jacobs L.F. (2020). How Ambient Environment Influences Olfactory Orientation in Search and Rescue Dogs. Chem. Senses.

[84] Cablk M.E., Sagebiel J.C., Heaton J.S., Valentin C. (2008). Olfaction-based Detection Distance: A Quantitative Analysis of How Far Away Dogs Recognize Tortoise Odor and Follow It to Source. Sensors.

[85] Gsell A., Innes J., De Monchy P., Brunton D. (2010). The success of using trained dogs to locate sparse rodents in pest-free sanctuaries. Wildl. Res..

[86] Jezierski T. (2011). Psy w Służbie Policji, Wojska i Ratownictwa. http://ph.ptz.icm.edu.pl/wp-content/uploads/2016/12/2-Jezierski.pdf.

[87] Rolland R.M., Hamilton P.K., Kraus S.D., Davenport B., Gillett R.M., Wasser S.K. (2006). Faecal sampling using detection dogs to study reproduction and health in North Atlantic right whales (*Eubalaena glacialis*). J. Cetacean Res. Manag..

[88] Karp D. (2020). Detecting small and cryptic animals by combining thermography and a wildlife detection dog. Sci. Rep..

[89] Reed S.E., Bidlack A.L., Hurt A., Getz W.M. (2011). Detection distance and environmental factors in conservation detection dog surveys. J. Wildl. Manag..

[90] Dahlgren D., Elmore R.D., Smith D.A., Hurt A., Arnett E.B., Connelly J.W. (2012). Use of Dogs in Wildlife Research and Management. Wildl. Tech. Man..

[91] Hussein A.K., Sullivan M., Penderis J. (2012). Effect of brachycephalic, mesaticephalic, and dolichocephalic head conformations on olfactory bulb angle and orientation in dogs as determined by use of in vivo magnetic resonance imaging. Am. J. Veter. Res..

[92] Meola S.D. (2013). Brachycephalic Airway Syndrome. Top. Companion Anim. Med..

[93] Hall N., Glenn K., Smith D.W., Wynne C.D.L. (2015). Performance of Pugs, German Shepherds, and Greyhounds (*Canis lupus familiaris*) on an odor-discrimination task. J. Comp. Psychol..

[94] Schoon G. (2005). The effect of the ageing of crime scene objects on the results of scent identification line-ups using trained dogs. Forensic Sci. Int..

[95] Williams M., Johnston J.M. (2002). Training and maintaining the performance of dogs (*Canis familiaris*) on an increasing number of odor discriminations in a controlled setting. Appl. Anim. Behav. Sci..

[96] Lazarowski L., Krichbaum S., DeGreeff L.E., Simon A., Singletary M., Angle C., Waggoner L.P. (2020). Methodological Considerations in Canine Olfactory Detection Research. Front. Veter. Sci..

[97] Rygg A.D., Van Valkenburgh B., A Craven B. (2017). The Influence of Sniffing on Airflow and Odorant Deposition in the Canine Nasal Cavity. Chem. Senses.

[98] Concha A., Mills D.S., Feugier A., Zulch H., Guest C., Harris R., Pike T.W. (2014). Using sniffing behavior to differentiate true negative from false negative responses in trained scent-detection dogs. Chem. Senses.

[99] Lasa J., Browarska-Walczowska A. (2003). Zmysł Węchu, Kryminalistyka, Metody Analityczne.

[100] Kokocinska-Kusiak A., Matalińska J., Sacharczuk M., Sobczyńska M., Góral-Radziszewska K., Wileńska B., Misicka A., Jezierski T. (2020). Can mice be trained to discriminate urine odor of conspecifics with melanoma before clinical symptoms appear?. J. Veter. Behav..

[101] Gazit I., Terkel J. (2003). Explosives detection by sniffer dogs following strenuous physical activity. Appl. Anim. Behav. Sci..

[102] Poling A., Weetjens B.J., Cox C., Beyene N., Bach H., Sully A. (2010). Teaching Giant African Pouched Rats to Find Landmines: Operant Conditioning with Real Consequences. Behav. Anal. Pract..

[103] McLean I. (2001). What the Dog’s Nose Knows. J. Mine Action.

[104] Phelan J.M. (2002). Chemical Sensing for Buried Landmines—Fundamental Processes Influencing Trace Chemical Detection.

[105] Phelan J., Webes S., McLea I.G. (2003). Chemical Sensing for Buried Landmines: Fundamental Processes Influencing Trace Chemical Detection. Mine Detection Dogs: Training Operations and Odour Detection.

[106] Fjellanger R., McLea I.G. (2003). The REST Concept. Mine Detection Dogs Training, Operations and Odour Detection.

[107] Hayter D., McLea I.G. (2003). Training dogs to detect tripwires. Mine Detection Dogs T raining, Operations and Odour Detection.

[108] Bach H., McLean I. (2003). Remote Explosive Scent Tracing: Genuine or a Paper Tiger?. J. Conv. Weapons Destr..

[109] Katz S.R., Midkiff C.R. (1998). Unconfirmed Canine Accelerant Detection: A Reliability Issue in Court. J. Forensic Sci..

[110] Kurz M.E., Billard M., Rettig M., Augustiniak J., Lange J., Larsen M., Warrick R., Mohns T., Bora R., Broadus K. (1994). Evaluation of canines for accelerant detection at fire scenes. J. Forensic Sci..

[111] Kurz M.E., Schultz S., Griffith J., Broadus K., Sparks J., Dabdoub G., Brock J. (1996). Effect of background interference on accelerant detection by canines. J. Forensic Sci..

[112] Tindall R., Lothridge K. (1995). An Evaluation of 42 Accelerant Detection Canine Teams. J. Forensic Sci..

[113] Arner L., Johnson G., Skovronek H. (1986). Delineating toxic areas by canine olfaction. J. Hazard. Mater..

[114] Crook A. (2000). Use of odour detection dogs in residue management programs. Asian-Australas. J. Anim. Sci..

[115] Adams G., Johnson K. (1994). Sleep, work, and the effects of shift work in drug detector dogs *Canis familiaris*. Appl. Anim. Behav. Sci..

[116] Lorenzo N., Wan T., Harper R.J., Hsu Y.-L., Chow M., Rose S., Furton K.G. (2003). Laboratory and field experiments used to identify Canis lupus var. familiaris active odor signature chemicals from drugs, explosives, and humans. Anal. Bioanal. Chem..

[117] Rouhi A.M. (1997). Detecting Illegal Substances. Chem. Eng. News Arch..

[118] Kalmus H. (1955). The discrimination by the nose of the dog of individual human odours and in particular of the odours of twins. Br. J. Anim. Behav..

[119] Schoon G., De Bruin J. (1994). The ability of dogs to recognize and cross-match human odours. Forensic Sci. Int..

[120] Settle R.H., Sommerville B.A., McCormick J., Broom D.M. (1994). Human scent matching using specially trained dogs. Anim. Behav..

[121] Schoon G. (1996). Scent identification lineups by dogs (*Canis familiaris*): Experimental design and forensic application. Appl. Anim. Behav. Sci..

[122] Harvey L.M., Harvey J.W. (2003). Reliability of bloodhounds in criminal investigations. J. Forensic Sci..

[123] Fenton V. (1992). The use of dogs in search, rescue and recovery. J. Wilderness Med..

[124] Hebard C. (1993). Use of search and rescue dogs. J. Am. Vet. Med. Assoc..

[125] Lasseter A.E., Jacobi K.P., Farley R., Hensel L. (2003). Cadaver dog and handler team capabilities in the recovery of buried human remains in the southeastern United States. J. Forensic Sci..

[126] Komar D. (1999). The use of cadaver dogs in locating scattered, scavenged human remains: Preliminary field test results. J. Forensic Sci..

[127] Engeman R.M., Rodriquez D.V., Linnell M.A., Pitzler M.E. (1998). A review of the case histories of the brown tree snakes (*Boiga irregularis*) located by detector dogs on Guam. Int. Biodeterior. Biodegrad..

[128] Engeman R.M., Vice D.S., Rodriguez D.V., Gruver K.S., Santos W.S., Pitzler M.E. (1998). Effectiveness of the detector dogs used for deterring the dispersal of Brown Tree Snakes. Pac. Conserv. Biol..

[129] Engeman R.M., Vice D.S., York D., Gruver K.S. (2002). Sustained evaluation of the effectiveness of detector dogs for locating brown tree snakes in cargo outbound from Guam. Int. Biodeterior. Biodegrad..

[130] Nakash J., Osem Y., Kehat M. (2000). A suggestion to use dogs for detecting red palm weevil (Rhynchophorus ferrugineus) infestation in date palms in Israel. Phytoparasitica.

[131] Wallner W.E., Ellis T.L. (1976). Olfactory Detection of Gypsy Moth Pheromone and Egg Masses by Domestic Canines. Environ. Entomol..

[132] Brooks S.E., Oi F.M., Koehler P.G. (2003). Ability of Canine Termite Detectors to Locate Live Termites and Discriminate Them from Non-Termite Material. J. Econ. Entomol..

[133] Culliney T., Grace J. (2000). Prospects for the biological control of subterranean termites (Isoptera: Rhinotermitidae), with special reference to *Coptotermes formosanus*. Bull. Entomol. Res..

[134] Lewis V., Fouche C.F., Lemaster R.L. (1997). Evaluation of dog-assisted searches and electronic odor devices for detecting the western subterranean termite. For. Prod. J..

[135] Welch J.B. (1990). A Detector Dog for Screwworms (Diptera: Calliphoridae). J. Econ. Entomol..

[136] Shelby R.A., Schrader K.K., Tucker A., Klesius P.H., Myers L.J. (2004). Detection of catfish off-flavour compounds by trained dogs. Aquac. Res..

[137] Hanson T.R. (2003). Economic Impact of Off-Flavor to the U.S. Catfish Industry. ACS Symposium Series.

[138] Kauhanen E., Harri M., Nevalainen A., Nevalainen T. (2002). Validity of detection of microbial growth in buildings by trained dogs. Environ. Int..

[139] Wasser S.K., Davenport B., Ramage E.R., E Hunt K., Parker M., Clarke C., Stenhouse G. (2004). Scat detection dogs in wildlife research and management: Application to grizzly and black bears in the Yellowhead Ecosystem, Alberta, Canada. Can. J. Zool..

[140] Kohn M.H., Wayne R.K. (1997). Facts from feces revisited. Trends Ecol. Evol..

[141] Mills L., Citta J.J., Lair K.P., Schwartz M.K., Tallmon D.A. (2000). Estimating Animal Abundance Using Noninvasive DNA Sampling: Promise and Pitfalls. Ecol. Appl..

[142] Wasser S.K., Hunt K.E., Brown J.L., Cooper K., Crockett C.M., Bechert U., Millspaugh J.J., Larson S., Monfort S.L. (2000). A Generalized Fecal Glucocorticoid Assay for Use in a Diverse Array of Nondomestic Mammalian and Avian Species. Gen. Comp. Endocrinol..

[143] Akenson J.J., Henjum M.G., Wertz T.L., Craddock T.J. (2001). Use of Dogs and Mark-Recapture Techniques to Estimate American Black Bear Density in Northeastern Oregon. Ursus.

[144] Smith D.A., Ralls K., Hurt A., Adams B., Parker M., Davenport B., Smith M.C., Maldonado J. (2003). Detection and accuracy rates of dogs trained to find scats of San Joaquin kit foxes (*Vulpes macrotis mutica*). Anim. Conserv..

[145] Smith D.A., Ralls K., Davenport B., Adams B., Maldonado J.E. (2001). Canine Assistants for Conservationists. Science.

[146] Kerley L.L., Salkina G.P. (2007). Using Scent-Matching Dogs to Identify Individual Amur Tigers from Scats. J. Wildl. Manag..

[147] Robert M., Laporte P. (1997). Field Techniques for Studying Breeding Yellow Rails (Técnicas de Campo para Estudiar a Individuos Reproductivos de Coturnicops Noveboracensis). J. Field Ornithol..

[148] Shute N. (1990). Dogging Rare Geese to Save Them. Natl. Wildl..

[149] Browne C.M. (2005). The Use of Dogs to Detect New Zealand Reptile Scents: A Thesis Presented in Partial Fulfilment of the Requirements for the Degree of Master of Science in Zoology at Massey University, Palmerston North, New Zealand. Master’s Thesis.

[150] Colbourne R. (1992). Little spotted kiwi (Apteryx owenii): Recruitment and behaviour of juveniles on Kapiti Island, New Zealand. J. R. Soc. N. Z..

[151] Dzieciol M., Niżański W., Ochota M., Kozdrowski R., Stańczyk E. (2012). Observation on Possibility to Identify by the Stud Dogs the Signs of the Fertile Period in Bitches. J. Anim. Veter. Adv..

[152] Dzięcioł M., Stańczyk E., Noszczyk-Nowak A., Niżański W., Ochota M., Kozdrowski R. (2012). Influence of bitches sex pheromones on the heart rate and other chosen parameters of blood flow in stud dogs (*Canis familiaris*). Res. Veter. Sci..

[153] Dzięcioł M., Niżański W., Stańczyk E., Kozdrowski R., Najder-Kozdrowska L., Twardoń J. (2013). The influence of antibiotic treatment of bitches in oestrus on their attractiveness to males during mating. Pol. J. Veter. Sci..

[154] Kiddy C.A., Mitchell D.S., Bolt D.J., Hawk H.W., Haney A.F., Schomberg D.W. (1978). Detection of Estrus-Related Odors in Cows by Trained Dogs. Biol. Reprod..

[155] Kiddy C., Mitchell D., Hawk H. (1984). Estrus-Related Odors in Body Fluids of Dairy Cows. J. Dairy Sci..

[156] Hawk H., Conley H., Kiddy C. (1984). Estrus-Related Odors in Milk Detected by Trained Dogs. J. Dairy Sci..

[157] D’Aniello B., Fierro B., Scandurra A., Pinelli C., Aria M., Semin G.R. (2021). Sex differences in the behavioral responses of dogs exposed to human chemosignals of fear and happiness. Anim. Cogn..

[158] Semin G.R., Scandurra A., Baragli P., Lanatà A., D’Aniello B. (2019). Inter- and Intra-Species Communication of Emotion: Chemosignals as the Neglected Medium. Animals.

[159] Bakeman U., Eilam H., Schild C.M., Grinstein D., Eshed Y., Laster M., Fride E., Anavi-Goffer S. (2019). Detection of Impending Aggressive Outbursts in Patients with Psychiatric Disorders: Violence Clues from Dogs. Sci. Rep..

[160] Buszewski B., Ligor T., Jezierski T., Wenda-Piesik A., Walczak M., Rudnicka J. (2012). Identification of volatile lung cancer markers by gas chromatography–mass spectrometry: Comparison with discrimination by canines. Anal. Bioanal. Chem..

[161] Angle C., Waggoner L.P., Ferrando A., Haney P., Passler T. (2016). Canine Detection of the Volatilome: A Review of Implications for Pathogen and Disease Detection. Front. Veter. Sci..

[162] Brown S.W., Goldstein L.H. (2011). Can seizure-alert dogs predict seizures?. Epilepsy Res..

[163] Catala A., Grandgeorge M., Schaff J.-L., Cousillas H., Hausberger M., Cattet J. (2019). Dogs demonstrate the existence of an epileptic seizure odour in humans. Sci. Rep..

[164] Dominguez-Ortega L., Díaz-Gállego E., Pozo F., García-Armenter S.C., Comino M.S., Dominguez-Sanchez E. (2013). Narcolepsy and odor: Preliminary report. SEMERGEN Med. De Fam..

[165] Wells D.L., Lawson S.W., Siriwardena A. (2008). Canine Responses to Hypoglycemia in Patients with Type 1 Diabetes. J. Altern. Complement. Med..

[166] Weber K.S., Roden M., Müssig K. (2015). Do dogs sense hypoglycaemia?. Diabet. Med..

[167] Reeve C., Cummings E., McLaughlin E., Smith S., Gadbois S. (2020). An Idiographic Investigation of Diabetic Alert Dogs’ Ability to Learn from a Small Sample of Breath Samples from People with Type 1 Diabetes. Can. J. Diabetes.

[168] Guest C., Pinder M., Doggett M., Squires C., Affara M., Kandeh B., Dewhirst S., Morant S.V., D’Alessandro U., Logan J.G. (2019). Trained dogs identify people with malaria parasites by their odour. Lancet Infect. Dis..

[169] McCulloch M., Jezierski T., Broffman M., Hubbard A., Turner K., Janecki T. (2006). Diagnostic Accuracy of Canine Scent Detection in Early- and Late-Stage Lung and Breast Cancers. Integr. Cancer Ther..

[170] Ehmann R., Boedeker E., Friedrich U., Sagert J., Dippon J., Friedel G., Walles T. (2012). Canine scent detection in the diagnosis of lung cancer: Revisiting a puzzling phenomenon. Eur. Respir. J..

[171] Jezierski T., Walczak M., Ligor T., Rudnicka J., Buszewski B. (2015). Study of the art: Canine olfaction used for cancer detection on the basis of breath odour. Perspectives and limitations. J. Breath Res..

[172] Walczak M., Jezierski T., Górecka-Bruzda A., Sobczyńska M., Ensminger J. (2012). Impact of individual training parameters and manner of taking breath odor samples on the reliability of canines as cancer screeners. J. Veter. Behav..

[173] Amundsen T., Sundstrøm S., Buvik T., Gederaas O.A., Haaverstad R. (2013). Can dogs smell lung cancer? First study using exhaled breath and urine screening in unselected patients with suspected lung cancer. Acta Oncol..

[174] Gordon R.T., Schatz C.B., Myers L.J., Kosty M., Gonczy C., Kroener J., Tran M., Kurtzhals P., Heath S., Koziol J.A. (2008). The Use of Canines in the Detection of Human Cancers. J. Altern. Complement. Med..

[175] Cornu J.-N., Cancel-Tassin G., Ondet V., Girardet C., Cussenot O. (2011). Olfactory Detection of Prostate Cancer by Dogs Sniffing Urine: A Step Forward in Early Diagnosis. Eur. Urol..

[176] Elliker K.R., A Sommerville B., Broom D.M., Neal D.E., Armstrong S., Williams H.C. (2014). Key considerations for the experimental training and evaluation of cancer odour detection dogs: Lessons learnt from a double-blind, controlled trial of prostate cancer detection. BMC Urol..

[177] Horvath G., Järverud G.A.K., Järverud S., Horváth I. (2008). Human Ovarian Carcinomas Detected by Specific Odor. Integr. Cancer Ther..

[178] Willis C.M., Church S.M., Guest C.M., Cook W.A., McCarthy N., Bransbury A.J., Church M.R.T., Church J.C.T. (2004). Olfactory detection of human bladder cancer by dogs: Proof of principle study. BMJ.

[179] Sonoda H., Kohnoe S., Yamazato T., Satoh Y., Morizono G., Shikata K., Morita M., Watanabe A., Kakeji Y., Inoue F. (2011). Colorectal cancer screening with odour material by canine scent detection. Gut.

[180] Williams H., Pembroke A. (1989). Sniffer Dogs in the Melanoma Clinic?. Lancet.

[181] Lamote K., Janssens E., Schillebeeckx E., Lapperre T.S., De Winter B.Y., Van Meerbeeck J.P. (2020). The scent of COVID-19: Viral (semi-)volatiles as fast diagnostic biomarkers?. J. Breath Res..

[182] Steppert C., Steppert I., Sterlacci W., Bollinger T. (2021). Rapid detection of SARS-CoV-2 infection by multicapillary column coupled ion mobility spectrometry (MCC-IMS) of breath. A proof of concept study. J. Breath Res..

[183] Jendrny P., Schulz C., Twele F., Meller S., Von Köckritz-Blickwede M., Osterhaus A.D.M.E., Ebbers J., Pilchová V., Pink I., Welte T. (2020). Scent dog identification of samples from COVID-19 patients—A pilot study. BMC Infect. Dis..

[184] Grandjean D., Sarkis R., Tourtier J.P., Julien-Lecocq C., Benard A., Roger V., Levesque E., Bernes-Luciani E., Maestracci B., Morvan P. (2020). Detection dogs as a help in the detection of COVID-19 Can the dog alert on COVID-19 positive persons by sniffing axillary sweat samples? Proof-of-concept study. PLoS ONE.

[185] Mendel J., Frank K., Edlin L., Hall K., Webb D., Mills J., Holness H.K., Furton K.G., Mills D. (2021). Preliminary accuracy of COVID-19 odor detection by canines and HS-SPME-GC-MS using exhaled breath samples. Forensic Sci. Int. Synerg..

[186] Aksenov A.A., Sandrock C.E., Zhao W., Sankaran S., Schivo M., Harper R., Cardona C.J., Xing Z., Davis C.E. (2014). Cellular Scent of Influenza Virus Infection. ChemBioChem.

[187] El Qader A.A., Lieberman D., Avni Y.S., Svobodin N., Lazarovitch T., Sagi O., Zeiri Y. (2015). Volatile organic compounds generated by cultures of bacteria and viruses associated with respiratory infections. Biomed. Chromatogr..

[188] Mashir A., Paschke K.M., Van Duin D., Shrestha N.K., Laskowski D., Storer M., Yen-Lieberman B., Gordon S.M., Aytekin M., A Dweik R. (2011). Effect of the influenza A (H1N1) live attenuated intranasal vaccine on nitric oxide (FE NO) and other volatiles in exhaled breath. J. Breath Res..

[189] Alasaad S., Permunian R., Gakuya F., Mutinda M., Soriguer R.C., Rossi L. (2012). Sarcoptic-mange detector dogs used to identify infected animals during outbreaks in wildlife. BMC Veter. Res..

[190] Richards K.M., Cotton S.J., Sandeman R.M. (2008). The use of detector dogs in the diagnosis of nematode infections in sheep feces. J. Veter. Behav..

[191] Jezierski T., Dzięcioł M., Szumny A., Niżański W., Woszczyło M., Pieczewska B., Godzińska E.J. (2018). Discrimination of estrus odor in urine by male dogs in different experimental settings. J. Veter. Behav..

[192] Samuel L., Arnesen C., Zedrosser A., Rosell F. (2020). Fears from the past? The innate ability of dogs to detect predator scents. Anim. Cogn..

[193] Hirano Y., Oosawa T., Tonosaki K. (2000). Electroencephalographic olfactometry (EEGO) analysis of odour responses in dogs. Res. Veter. Sci..

[194] Prichard A., Chhibber R., King J., Athanassiades K., Spivak M., Berns G.S. (2020). Decoding Odor Mixtures in the Dog Brain: An Awake fMRI Study. Chem. Senses.

[195] Szabó D., Czeibert K., Kettinger Á., Gácsi M., Andics A., Miklosi A., Kubinyi E. (2019). Resting-state fMRI data of awake dogs (*Canis familiaris*) via group-level independent component analysis reveal multiple, spatially distributed resting-state networks. Sci. Rep..

[196] Dzięcioł M. Influence of human putative sex pheromones on brainactivity monitored by rsfMRI. Proceedings of the Dog Olfactory Conference, Worldwide Online Conference.

[197] Lorig T.S. (2012). Beyond Self-report: Brain Imaging at the Threshold of Odor Perception. Chemosens. Percept..

[198] Polgár Z., Miklósi Á., Gácsi M. (2015). Strategies Used by Pet Dogs for Solving Olfaction-Based Problems at Various Distances. PLoS ONE.

[199] Hare B., Tomasello M. (1999). Domestic dogs (*Canis familiaris*) use human and conspecific social cues to locate hidden food. J. Comp. Psychol..

[200] Szetei V., Miklósi Á., Topál J., Csányi V. (2003). When dogs seem to lose their nose: An investigation on the use of visual and olfactory cues in communicative context between dog and owner. Appl. Anim. Behav. Sci..

[201] Bomers M.K., A Van Agtmael M., Luik H., Van Veen M.C., E Vandenbroucke-Grauls C.M.J., Smulders Y.M. (2012). Using a dog’s superior olfactory sensitivity to identify Clostridium difficile in stools and patients: Proof of principle study. BMJ.

[202] Los E.A., Ramsey K.L., Guttmann-Bauman I., Ahmann A.J. (2016). Reliability of Trained Dogs to Alert to Hypoglycemia in Patients with Type 1 Diabetes. J. Diabetes Sci. Technol..

[203] Gonder-Frederick L.A., Grabman J.H., Shepard J.A. (2017). Diabetes Alert Dogs (DADs): An assessment of accuracy and implications. Diabetes Res. Clin. Pract..

[204] Lippi G., Plebani M. (2019). Diabetes alert dogs: A narrative critical overview. Clin. Chem. Lab. Med..

[205] Hall N.J., Wynne C.D. (2018). Odor mixture training enhances dogs’ olfactory detection of Home-Made Explosive precursors. Heliyon.

[206] Dorman D., Foster M., Lazarowski L. (2021). Training with Multiple Structurally Related Odorants Fails to Improve Generalization of Ammonium Nitrate Detection in Domesticated Dogs (*Canis familiaris*). Animals.

[207] Lazarowski L., Dorman D.C. (2014). Explosives detection by military working dogs: Olfactory generalization from components to mixtures. Appl. Anim. Behav. Sci..

[208] Johnen D., Heuwieser W., Fischer-Tenhagen C. (2013). Canine scent detection—Fact or fiction?. Appl. Anim. Behav. Sci..

[209] Hayes J., McGreevy P., Forbes S., Laing G., Stuetz R. (2018). Critical review of dog detection and the influences of physiology, training, and analytical methodologies. Talanta.

[210] LitJulie L., Schweitzer J.B., Oberbauer A.M. (2011). Handler beliefs affect scent detection dog outcomes. Anim. Cogn..

[211] Johnen D., Heuwieser W., Fischer-Tenhagen C. (2017). An approach to identify bias in scent detection dog testing. Appl. Anim. Behav. Sci..

[212] Edwards T.L. (2019). Automated Canine Scent-Detection Apparatus: Technical Description and Training Outcomes. Chem. Senses.

[213] Hackner K., Pleil J. (2017). Canine olfaction as an alternative to analytical instruments for disease diagnosis: Understanding ‘dog personality’ to achieve reproducible results. J. Breath Res..

